# The potential of bacteriophage cocktail in eliminating Methicillin-resistant *Staphylococcus aureus* biofilms in terms of different extracellular matrices expressed by *PIA*, *ciaA*-*D* and *FnBPA* genes

**DOI:** 10.1186/s12941-015-0106-0

**Published:** 2015-11-11

**Authors:** Ahmed Sahib Abdulamir, Sabah A. A. Jassim, Rand R. Hafidh, Fatimah Abu Bakar

**Affiliations:** Microbiology Department, College of Medicine, Alnahrain University, 14222 Baghdad, Iraq; Applied Bio Research Inc., Windsor, Canada; Environmental Engineering, University of Windsor, Windsor, Canada; Microbiology Department, College of Medicine, Baghdad University, Baghdad, Iraq; Faculty of Food Science, University Putra Malaysia, Serdang, 43400 Selangor, Malaysia

**Keywords:** Methicillin-resistant *Staphylococcus aureus*, MRSA, MSSA, Biofilm, Bacteriophages, PIA, FnBPA

## Abstract

**Background:**

This study assessed novel approach of using highly lytic phages against methicillin-susceptible *Staphylococcus aureus* (MSSA) and methicillin-resistant *Staphylococcus aureus* (MRSA) biofilms with and without biofilm extracellular matrix- disrupting chemical.

**Method:**

The resultant phage-based control was assessed in relation to the type of biofilm extracellular matrix namely, polysaccharide intercellular adhesion (PIA) or proteinacious fibronectin-binding protein A (FnBPA). The biofilms were formed in vitro by 24 h incubation of bacteria in 96 wells microtiter plates at room temperature. The formed biofilms were assessed by tissue culture plate (TCP). Moreover, the nature of the biofilm was assessed by scanning electron microscopy (SEM) and PCR assay for detecting PIA genes, *ciaA*-*D* and FnBPA genes.

**Results:**

this study showed that applied phages with 0.08 % benezenthonium chloride, for PIA biofilms, and 0.06 % ethanol, for proteinacious FnBPA biofilms, exerted 100 % eradication for MSSA biofilms and about 78 % of MRSA biofilms. The phage-based control of biofilms with chemical adjuvant showed significantly higher efficiency than that without adjuvant (P < 0.05). Moreover, FnBPA biofilms were more common in MRSA than in MSSA while PIA biofilms were more common in MSSA than in MRSA. And the most resistant type of biofilms to phage-based control was FnBPA in MRSA where 50 % of biofilms were reduced but not eradicated completely.

**Conclusions:**

It is concluded that PIA-disturbing agent and protein denaturing alcohol can increase the efficiency of attacking phages in accessing host cell walls and lysing them which in turn lead to much more efficient MRSA and MSSA biofilm treatment and prevention.

## Background

S*taphylococcus aureus* (*S. aureus*) is the prime cause of hospital-associated infections. According to antibiotic resistance profiling, S*taphylococcus aureus* is divided into Methicillin-susceptible *S. aureus* (MSSA) and Methicillin-resistant *S. aureus* (MRSA) which represents an especially big threat to the public-health system [1]. Traditionally, MRSA infections have been limited to hospitals and predisposed individuals, such as the elderly, immunocompromised and patients undergoing surgery [2, 3]. More recently, however, MRSA infections have occurred outside of the hospital setting [4]. Community-associated (CA)-MRSA were first reported in the Northern USA, where they caused fatal infections in otherwise healthy children [5]. Later, CA-MRSA has become a global problem, with the most serious epidemic seen in the USA [6]. According to recent estimates, MRSA infections cause more deaths annually in the USA than HIV/AIDS [7].

Staphylococci are usually related to infections of implanted medical devices. The predominant species isolated in these infections are *Staphylococcus epidermidis* and *Staphylococcus aureus.* Moreover, most of the attached *S. aureus* are in fact MRSA. The main virulent factor of these bacteria is the ability to form biofilm on polymeric surfaces to which it adheres and colonizes artificial materials [4, 8]. Biofilms are a population of multilayered cells growing on a surface and enclosed in exopolysaccharide matrix. Biofilm formations are considered to be a four step process: (a) attachment, (b) colonization and multiplication, (c) maturation and (d) dispersion.

Antibiotic resistance and biofilm-forming capacity contribute to the success of Staphylococcus aureus as a human pathogen in both healthcare and community settings [9]. The biofilm matrix is a complex mixture of macromolecules, including exopolysaccharides, proteins, and DNA. These virulence factors do not function independently of each other and the biofilm phenotype expressed by clinical isolates of *S. aureus* is influenced by acquisition of the methicillin resistance gene mecA. Methicillin-sensitive *S. aureus* strains commonly produce an icaADBC operon-encoded polysaccharide intercellular adhesin (PIA)-dependent biofilm. In contrast, the release of extracellular DNA (eDNA) and cell surface expression of a number of sortase-anchored proteins, and the major autolysin have been implicated in the biofilm phenotype of MRSA isolates [9]. The main exopolysaccharide of the SA biofilm matrix is a polymer of poly-*N*-acetylglucosamine, termed polysaccharide intercellular adhesin (PIA) or poly-*N*-acetylglucosamine (PNAG), whose synthesis depends on the enzymes encoded by the *icaADBC* operon in ica locus [10, 11]. Among ica genes, *icaA* and *icaD* have been reported to play a significant role in biofilm formation in MSSA and MRSA [12, 13].

However, the presence of PIA/PNAG exopolysaccharide is not found in all biofilms of MSSA or MRSA strains. Several studies have uncovered the existence of *S. aureus* isolates able to produce alternative biofilm matrixes [14–16]. When this occurs, it appears that proteins usually take the responsibility for mediating cell-to-cell interactions and multicellular behavior. More recently, two independent research groups have described a novel *S. aureus* biofilm phenotype mediated by the fibronectin-binding proteins, FnBPA and FnBPB, being FnBPA is more important and more constantly associated with proteinacious biofilm of MSSA or MRSA [17, 18]. Therefore, there are two types of biofilms for MSSA and MRSA bacteria, polysaccharide- or PIA- based biofilm and proteinacious, or FnBPA-based, biofilms. Nevertheless, the behaviour of these two types of biofilms was not studied well.

The rate at which *S. aureus* can develop or acquire resistance to new antibiotics seems to be higher than the rate at which new antibiotics are discovered and developed. Accordingly, new control measures, rather than antibiotics, are needed in action to contain the increasing hazard of MRSA [19]. Moreover, there is a need to a safe, widely accepted, and revolutionary measures for the control and therapy of MRSA. Worldwide, an estimated 2 billion people carry some form of *S. aureus*; of these, up to 53 million (2.7 % of carriers) are thought to carry MRSA [20]. One of the promising new measures of MRSA control is bacteriophages (phages). Phages are present abundantly in the environment and humans ingest billions of phages daily without harm; therefore, they are considered safe for humans and animals [21].

However, the most challenging problem for MRSA control, either using phages or other agents, is their ability to produce highly protective biofilms. Biofilms of MRSA are considered the main source for providing virulent bacterial cells ready to spread infection in hospitals and in community. Therefore, in our opinion, there is a need to formulate an effective non-antibiotic phage-based control for MRSA and MSSA biofilms. In the current study, anti-MSSA and anti-MRSA phages were optimized and designed and were used to assess phage control to MSSA and MRSA biofilms. Moreover, the nature of intercellular adhesion of MSSA and MRSA biofilms, PIA or FnBPA-based biofilms, were studied and their response to phage-based control was accordingly evaluated.

## Methods

### Sampling of bacteria

Seven isolates of MRSA and 10 isolates of MSSA were chosen to be used in the current study. These isolates were collected, as part of the routine hospital work, from clinical wound samples in three hospitals in Slenagor state in Malaysia, Hospital Serdang, Hospital Kajang and Hospital HUKL using Microbact GNB 12A system (Oxoid, UK) with 99 % confirmatory rate. The diagnosed *S. aureus* bacteria were then subjected to a series of selective and differential media to identify MRSA bacteria which are the most important target of this study. The current study has been approved by the ethical committee of University Putra Malaysia.

### Isolation, identification, and propagation of MSSA and MRSA

Bacteria were swabbed from infected regions and were inoculated into nutrient broth (NB) (Merck, Germany) for 18 h at 37 °C. Next day, grown bacteria in NB were ABC streaked onto nutrient agar (NA) (Merck, Germany) and incubated for 18 h at 37 °C. Next day, different colonies were grown, the suspected colonies of *S. aureus* were streaked onto a selective and differential medium namely, Mannitol salt agar (MSA) (Merck, Germany) for 18 h at 35 °C. Next day, the grown bacteria in yellow colonies were confirmed as *S. aureus*. In addition, light microscopy was used as a supportive tool for *S. aureus* diagnosis. In order to identify MRSA strains, MRSA chromogenic agar or MRSA agar (Merck, Germany) was used. MRSA agar is composed of 11 g peptone mixture, 1.9 g chromogenic substrate, 78 g growth factors and 12.5 biological agar. In addition, MRSA agar was used with added oxacillin (6 mg/L) (Sigma, USA) in the preparation of MRSA agar. MRSA agar was used as a screening medium for the determination of methicillin resistance and oxacillin resistance in *Staphylococcus aureus.* The presence of growth indicated oxacillin and methicillin resistance. Lack of growth indicated that the strain lacks the *mec*A resistance gene. Only the non-inhibited colonies in MRSA selective agar were considered MRSA.

### Reference strains of MRSA and MSSA

Reference strains of MRSA and MSSA were involved in the current study along with the isolated and identified clinical samples. Two reference strains of MRSA were used namely, ATCC 700699 and ATCC 43300 and two MSSA reference strains were used, namely, ATCC 11987 and ATCC 27691.

### The strategy of the current study

First, the biofilm formation is compared between MSSA and MRSA bacteria. Second, the type of biofilms produced are assessed and categorized into PIA-based biofilms or proteinacious FnBPA-based biofilms in both MSSA and MRSA using SEM and PCR detection for *icaA*-*D* genes, which are responsible for PIA synthesis, and *FnBPA* gene, which is responsible for fibronectin-binding protein synthesis. Third, the MSSA and MRSA biofilms are challenged with high titre of specific lytic phages for disintegrating the biofilm matrix and lysing the integrated bacteria. Fourth, the effectiveness of the phage-based control for MRSA and MSSA biofilms is assessed in comparison with MSSA versus MRSA and PIA-based versus FnBPA-based biofilms. In addition, the augmentation of the efficiency of phage-based control was assessed by coupling attacking phages with PIA-disturbing or protein-denaturing chemical adjuvants.

#### Isolation of wild phages to MSSA and MRSA

Phages to MSSA and MRSA were isolated from 3 different sources; Hospital environmental dirt, sewage disposal, and cattle waste. Crude specimens of approximately 50 g of environmental dirt, sewage disposal, or cattle waste were each collected in a sterile sample collection tube (100 ml). The procedure pursued was done according to [22] with some modification. One to three grams of each specimen were transferred into 90 ml of nutrient broth (NB) and vortexed for 30 s. Then 1 ml of 8 h NB cultures of the target MRSA clinical isolate or reference strains was added and incubated at 37 °C. After 18 h, 10 ml of the mixture were withdrawn into a sterile 15 ml test tube and centrifuged for 5 min at 5000×*g* at room temperature. Supernatant was aspirated into new sterile 15 ml test tubes. To the supernatant, 1 ml of chloroform (Sigma, USA) was added with gentle shaking of tubes for 5 min then all tubes were incubated on crushed ice for 5 min. A milky solution appears due to bacterial proteins digestion by chloroform. Centrifugation at 5000×*g* for 5 min at room temperature was carried-out. Top aqueous supernatant was collected into 15 ml sterile tube and stored at 4 °C as a possible phage solution.

#### Testing for the presence of wild phages (phage spot lysis assay)

Thin bacterial lawns of clinical isolates or reference strains were prepared by adding 500 µl of NB 18 h cultures on NA plates, allowing the liquid to soak into the plate. The plates were kept for 1 h at room temperature. Ten µl of the prepared phage suspension were spotted on bacterial lawns and incubated at 37 C. Plaques or lysis spot were observed after 6 h and 18 h. The detection of phage presence was based on visual appearance of lysis zone. Positive results were expressed by either clear or semi-clear (turbid) lysis zone while negative results were expressed by the absence of such lysis zones [23].

#### The approach of phage isolation and propagation

A series of optimization steps were used in order to augment the efficacy of phage hunting/isolation techniques. The optimization manoeuvres that were taken into account are:

The collection of crude phage samples was diversified in a way that 1 g of as minimal as 10 different samples from hospital dirt, sewage, and cattle waste was used to form the crude mixture. Samples of crude mixture (10 g) were placed in 100 ml Erlenmeyer flask with cotton-plugged filled with 80 ml of NB. Then, 10 MRSA isolates were used together to inoculate the phage mixture with 1 ml from each 18 h MRSA culture. After 18 h of incubation at 37 °C, 10 ml were dispensed into a sterile 15-mL plastic culture tubes. After centrifugation at 5000×*g* for 5 min at room temperature, the supernatant was transferred into 1.5 ml sterile microcentrifuge. Then 1:10 chloroform to lysate ratio was added with gentle shaking for 5 min at room temperature in order to lyse the bacterial cells followed by further 3 min incubation in crushed ice. The mixture was then centrifuged 5000×*g* for 15 min at room temperature and the supernatant was transferred into a 1.5 ml sterile microcentrifuge tubes. To this step, the isolated phages mixture was produced. The produced mixture of the isolated phages was propagated on the desired target bacteria lawn as it is earlier mentioned in the procedure of the phage spot lysis test [23].

#### Production of the transient phage stock

The produced mixture of the isolated phages was propagated on each target bacterial lawn as it is earlier mentioned in the procedure of the phage spot lysis assay. Lysis zones, if any, were cut by a sterile scalpel and plunged into 300 µl of Lambda buffer in 1.5 ml sterile microcentrifuge tubes for 20 min with intermittent gentle shaking. 1:10 chloroform to lysate ratio was added with gentle shaking for 5 min at room temperature in order to elute the phages from the agar and to lyse the bacterial cells. After further 3 min incubation in crushed ice the mixture centrifuged at 5000×*g* for 15 min at room temperature and the supernatant transferred in a 1.5 ml sterile microcentrifuge tubes [22].

### Biofilm formation

The MRSA and MSSA biofilm formation assays were performed as described [24]. Sterile 96-well polystyrene microtiter plates were used throughout the study. Each assay was performed in triplicate and repeated at least three times. Briefly, bacterial isolates from fresh agar plates were inoculated in nutrient broth and incubated for 24 h at 37 °C in stationary condition and diluted (1 in 50) with fresh medium. Individual wells of sterile, polystyrene, flat-bottom tissue culture plates were filled with 200 μl aliquots of the diluted cultures (10^8^ cfu/ml). The negative control was composed of broth only to check sterility and non-specific binding of media. The tissue culture plates were incubated for 24 h at 37 °C. After incubation, the content of each well was gently removed by tapping the plates. The wells were washed three times with 200 μl of phosphate buffer saline (PBS pH 7.2) to remove non-attached floating bacteria.

### Detection of biofilm formation by tissue culture plate method (TCP)

The TCP assay is most widely used and was considered as standard test for the detection of biofilm formation. All isolates were screened for their ability to form biofilm by the TCP method as described by Christensen et al. [25] with a modification in duration of incubation which was extended to 24 h, according to O’Toole and Kolter [26]. After the formation of the biofilm in tissue culture wells, 25 μl of 1 % solution of crystal violet were added to each well (this dye stains the cells but not the polystyrene) plates. The plates were incubated at room temperature for 15 min, rinsed thoroughly and repeatedly with water. Adherent cells, which usually formed biofilm on all side wells, were uniformly stained with crystal violet. Crystal violet-stained biofilm was solubilized in 200 μl of ethanol-acetone (80:20, vol/vol) (Merck, Germany). Afterwards, 100 μl were transferred to a new polystyrene microtiter dish which was subjected to micro ELISA auto reader at 570 nm wavelength. Optical densities (OD) of stained adherent bacteria were determined and were considered as indices for bacterial potential to adhere to surfaces and form biofilms. Experiments for each strain were performed in triplicate and repeated three times. To compensate for background absorbance, OD readings of wells with ethanol were used as blank and subtracted from all tests’ values. Biofilm production is considered high, moderate, weak, or zero according to the measurements of OD570 nm as shown in Table 1.

**Table 1:** Classification of biofilms formation according to TCP method [8]

Biofilm formation	Mean OD values
Zero	<0.05
Weak	0.05–0.12
Moderate	0.12–0.24
High	>0.24

### Phage application on MSSA and MRSA biofilms

Optimized and isolated phages to MSSA, 12 phages, and MRSA, 9 phages, in lambda buffer were applied on the formed biofilms of 10 clinical isolates and two reference strains of MSSA and 7 clinical isolates and 2 reference strains of MRSA. Ten (10) μl of anti-SA or anti-MRSA 10^12^ PFU/ml phages were applied on the formed biofilms in the tissue culture plates with and without adjuvant substances. Sublethal concentrations of chemical adjuvants together with applied phages were used in order to increase the permeability of the matrix of the biofilm as well as to increase the exposure of phage-binding receptors on the cell walls of MSSA and MRSA bacteria. For PIA type biofilms, after numerous trials and errors using a panel of PIA/PNAG-disturbing chemicals [data not shown], a sublethal dose, 0.08 %, of Benzethonium chloride was found to be the best in disintegrating PIA type biofilms when is mixed with applied phages; however, this dose was of no effect on either biofilms or phages when Benzethonium chloride was used alone. For FnBPA type biofilms, after numerous trial and error steps using a panel of protein-denaturing chemicals, sublethal dose, 0.06 %, of ethanol was found to be the best in disintegrating the proteinacious matrix of FnBPA type biofilms when is mixed with applied phages [data not shown]; however, this dose was of no effect on biofilms or phages when ethanol was used alone. Assessment of biofilm formation was conducted by using TCP method. The phage-based reduction of biofilm formation was scored from 0 to 3.

It is noteworthy to mention that plates were kept, without cover, stationary up to 24 h at room temperature. Microtiter plates were kept at room temperature without lid cover in order to mimic the sterilization conditions in hospitals where the sterilizing agents dry up after 30 min.

### Scanning Electron Microscopy (SEM)

Formed biofilms of MSSA and MRSA were fixed in 2.5 % (vol/vol) glutaraldehyde (Sigma, USA) in Dulbecco PBS (PH 7.2) for 1.5 h, rinsed with PBS, and then dehydrated through an ethanol series. Samples were dried and gold–palladium coated. SEM examinations were made on a JSM-840 SEM (JEOL Ltd., Tokyo, Japan) [27]. SEM was used in the current study for visualizing the formed biofilms and most importantly for assessing visually the type of the formed biofilms whether polysaccharide- or protein- based biofilm. The IPA-based biofilm appears usually with rough crystallized surface while FnBPA-based biofilm appears as smooth non-crystallized surface.

### PCR for the detection of *icaA*, *icaD,* and FnBPA genes

#### Bacterial DNA extraction

Chromosomal and plasmid DNA of the MSSA and MRSA bacteria were extracted using Wizard^®^ Genomic DNA Purification Kit with accessory reagents (Promega, USA); the method of extraction was done according to the manufacturer’s instructions. The obtained genomic DNA was rehydrated by adding 100 µl DNA Rehydration solution. Extracted DNA 260/280 nm ratio was >1.8 and quality was assessed by gel electrophoresis. Then, a 5 μl aliquot was used as a template for PCR amplification.

#### PCR protocols

The primers used were synthesized by the laboratories of Biotechnology institute of Malaysia. For *icaA*-*D* and FnBPA, PCR master mixture consisted of 2 µl of 10× PCR buffer, 0.8 µl of a 10 mM deoxynucleoside triphosphate mixture, 0.7 µl of forward and reverse primers at concentration of 10 pmol/µl, 1.5 µl of 25 mM MgCl2, and 0.2 µl of 0.5 U Taq DNA polymerase, and 50 ng of template DNA; the remaining volume consisted of distilled water. The PCR reaction was conducted in a thermocycler PCR system (PTC-110TM Model, MJ Research, Inc., USA). The PCR conditions, primres sequences are shown in Table 2.

**Table 2:** PCR conditions, primer sequences, and PCR products of *icaA*, *icaD*, and *FnBPA* genes

The gene	Primers sequences	PCR product (bp)	Reference	Thermal cycles
*icaA*	F-5′-GAC CTC GAA GTC AAT AGA GGT A R-5′-CCC AGT ATA ACG TTG GAT ACC	814	[28]	95 °C for 5 min, 35 cycles 94 °C 30 s, 59.5 °C 30 s, 72 °C 50 s, and 72 °C 5 min after conclusion of the 35 cycles [29]
*icaD*	F-5′-AGG CAA TAT CCA ACG GTA A R-5′-GTC ACG ACC TTT CTT ATA TT	371		
FnBPA	F-5′-AGGGATCCGATGGTGGAGGTGGATA-3′ R-5′-AGCCCGGGTGGCGTTGGTGGCACGATTGGAG	1274	[30]	95 °C 5 min, 35 cycles 94 °C 30 s, 65.5 °C 30 s, 72 °C 1 min and 72 °C 5 min after conclusion of the 35 cycles [30]

Afterwards, 5 μl of PCR products, *icaA*, *icaD* and FnBPA, were electrophoresed on 2 % agarose gel using QIAGEN GelPilot DNA Molecular Weight Marker as a ladder. PCR products were separated by an electrophoresis system at a constant voltage of 80 V for 50 min and were stained with 0.25 µg/ml ethidium bromide (Sigma, USA). PCR products were photographed under UV transilluminator (Vilber Lourmat, Cedex, France) and the photos were taken using gel documentation system, Bio Rad Gel Doc 2000 Model Imaging System (Bio-Rad, USA). The resulted bands of the PCR product for FnBPA, *icaA*, and *icaD* were expressed as positive and negative expression.

### Statistical analysis

Data of the current study was analyzed by SPSS version 12.0.1. For qualitative nominal data, Fisher exacts test was used. For quantitative nonparametric data, Mann–Whitney test was used. P values less than 0.05 was considered significant.

## Results

### Biofilm formation

All tested MRSA and MSSA isolates and reference strains developed various levels of biofilm expressed as weak, moderate, and high. The tested MSSA and MRSA isolates and reference strains showed variable tendencies for biofilm formation using TCP method (Table 3). In order to compare the level of biofilm formation between MSSA and MRSA, high/moderate versus weak groups of biofilm production was assessed in MSSA versus MRSA. Although tested MRSA showed higher tendency for biofilm production, no significant difference was found in biofilm production level between SA and MRSA (P = 0.36).

**Table 3:** The frequency of MRSA and MSSA isolates/reference strains in forming different levels of biofilm

Bacteria	High, N (%)	Moderate, N (%)	Weak, N (%)
MRSA	4 (44.4)	3 (33.3)	2 (22.2)
MSSA	3 (25)	3 (25)	6 (50)

### Types of biofilms produced

**Fig. 1:**
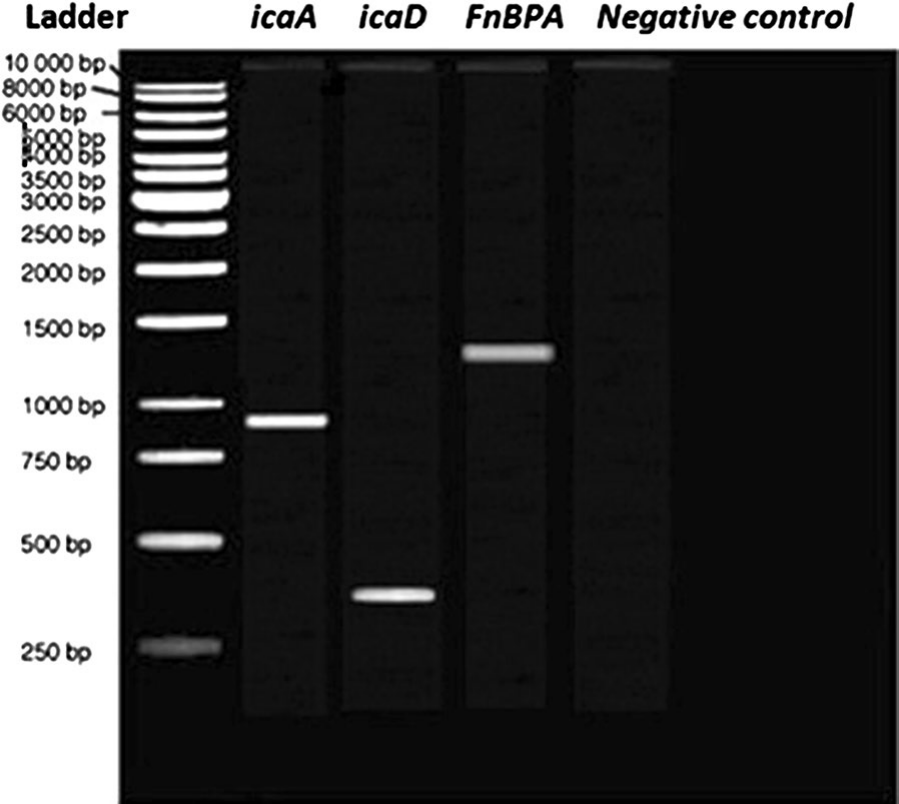
Gel electrogram for the PCR-based positive detection of *icaA*, *icaD*, and *FnBPA* genes in MRSA and MSSA bacteria

**Fig. 2:**
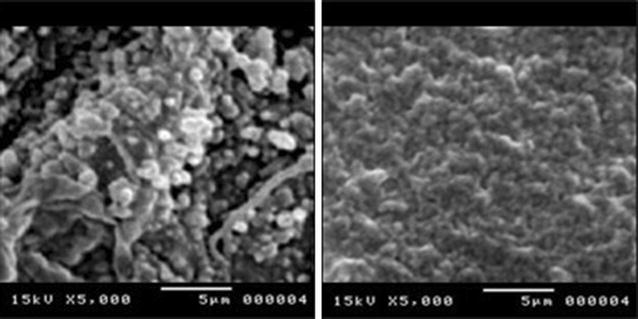
SEM photos for (*left*) PIA/PNAG crystalline rough surface *cagA*-*D*-positive biofilm of MRSA and (*right*) smooth proteinacious FnBPA-positive biofilm of MRSA

The type of biofilms produced was assessed according to the PCR-based detection of PIA genes, *ica* A and D, or FnBPA gene (Fig. 1). In addition, SEM screening was used to visualize the morphology of the produced biofilm and to help assess the type whether PIA- or FnBPA- based biofilm (Fig. 2). The results of both SEM and PCR were in harmony. The PIA, rough crystalline morphology as shown in SEM, was congruous with the positive detection of both genes, *icaA* and *icaD* while the smooth proteinacious morphology of the produced biofilms was congruous with the positive detection of FnBPA gene. However, only one MRSA isolate showed simultaneous positivity for both *icaA*-*D* and FnBPA genes but it was seen in SEM as smooth-surfaced proteinacious biofilm; therefore, it was categorized as FnBPA-based biofilm producing bacteria (Table 4).

**Table 4:** The type of produced biofilms in MRSA and MSSA bacteria assessed by SEM and PCR along with their corresponding level of biofilm production

Bacteria	PIA-based biofilm via PCR and rough crystalline biofilm via SEM			FnBPA-based biofilm via PCR and smooth proteinacious biofilm via SEM		
	High, N (%)	Moderate, N (%)	Weak, N (%)	High, N (%)	Moderate, N (%)	Weak, N (%)
MRSA	1 (11.1)	1 (11.1)	1 (11.1)	3 (33.3)	2 (22.2)	1 (11.1)
MSSA	2 (16.6)	2 (16.6)	4 (33.3)	1 (8.3)	1 (8.3)	2 (16.6)

According to Table 3, MRSA showed higher tendency to produce FnBPA type biofilms; 6 isolates with FnBPA type biofilms and 3 isolates with PIA type biofilms at ratio 2: 1. On the contrary, SA showed higher tendency to produce PIA type biofilms, 8 isolates, FnBPA type biofilm, 4 isolates at ratio 2: 1. This important finding might provide evidence for the different responses of MRSA and MSSA biofilms to antibiotics, antiseptics, or biological antibacterial agents such as bacteriophages which were used in the current study. In addition, in MRSA, FnBPA biofilm type was found to be mainly of high/moderate score while PIA- biofilm type was mainly of weak score of biofilm formation; however, these differences did not reach statistical significance in the level of biofilm production (high/moderate versus weak) between PIA and FnBPA types of biofilms in both MSSA and MRSA bacteria (P = 0.65).

### Phage-based biocontrol for MRSA and SA biofilms

Two sets of phage application experiments were assessed, phage application with and without chemical adjuvant on both types of biofilms, namely PIA and FnBPA in MRSA and MSSA bacteria. The magnitude of phage-based reduction of biofilm was measured as reduction score which was from 0 to 3. For both types of biofilms in both MSSA and MRSA bacteria, it was found that phage application reduced effectively the formed biofilms (Table 5). By using phages alone without adjuvant chemical, 6/8 (75 %), 1/4 (25 %), 1/3 (33.3 %), and 1/6 (16.6 %) of biofilms were reduced one score in MSSA PIA, MSSA FnBPA, MRSA PIA, and MRSA FnBPA, respectively (Table 5). The current findings revealed that phage-based reduction is more remarkable in MSSA than in MRSA and in PIA than in FnBPA. Accordingly, MRSA FnBPA group was the least responsive to phage-based reduction of biofilms when phages without chemical adjuvant were used. On the other hand, by using phages with chemical adjuvant, 100 % of biofilms, whether MSSA or MRSA, were reduced at least one score. Moreover, 3/8 (37.5 %), 2/4 (50 %), 2/3 (66.6 %), and 3/6 (50 %) of biofilms were reduced two score and more in MSSA PIA, MSSA FnBPA, MRSA PIA, and MRSA FnBPA, respectively (Table 5). Hence, it appears clearly that MRSA biofilms responded to the addition of the chemical adjuvant to the applied phages more obviously than MSSA biofilms.

**Table 5:** Phage-based reduction of biofilms, with or without using adjuvant chemical, in PIA and FnBPA types of both MRSA and SA

Bacteria	Type of biofilm	Phage application without chemical adjuvant	Phage application with chemical adjuvant
		Level of biofilm before: after (score of reduction)	Level of biofilm before: after (score of reduction)
SA-1	PIA	High: high (0)	High: weak (2)
SA-2	PIA	Mod: weak (1)	Mod: zero (2)
SA-3	PIA	weak: zero (1)	weak: zero (1)
SA-10	PIA	weak: zero (1)	weak: zero (1)
SA-5	PIA	High: High (0)	High: weak (2)
SA-6	PIA	weak: zero (1)	weak: zero (1)
ATCC 11987	PIA	Mod: weak (1)	Mod: weak (1)
SA-8	PIA	weak: zero (1)	weak: zero (1)
*Average score of reduction*		0.75	1.38
SA-9	FnBPA	Mod: Mod (0)	Mod: zero (2)
SA-4	FnBPA	High: high (0)	High: weak (2)
SA-7	FnBPA	weak: weak (0)	weak: zero (1)
ATCC 27691	FnBPA	weak: zero (1)	weak: zero (1)
*Average score of reduction*		0.25	1.5
MR-4	PIA	Mod: Mod (0)	Mod: zero (2)
MR-5	PIA	High: High (0)	High: weak (2)
MR-7	PIA	weak: zero (1)	weak: zero (1)
*Average score of reduction*		0.33	1.67
MR-1	FnBPA	High: high (0)	High: zero (3)
MR-2	FnBPA	Mod: mod (0)	Mod: weak (1)
MR-3	FnBPA	High: high (0)	High: weak (2)
MR-6	FnBPA	weak: zero (1)	weak: zero (1)
ATCC 700699	FnBPA	Mod: Mod (0)	Mod: zero (2)
ATCC 43300	FnBPA	High: high (0)	High: mod (1)
*Average score of reduction*		0.17	1.67

*Mod* represents moderate production of biofilms, *Zero* describes mean OD values of TCP method less than 0.01

By using Mann–Whitney test for comparing the median scores of phage reduction of biofilms among different groups of the study, it was found that the median reduction score by phages with chemical adjuvant was higher than by counterpart phages without chemical adjuvant in biofilms of MSSA PIA (P = 0.034), MSSA FnBPA (P = 0.047), MRSA PIA (P = 0.11), and MRSA FnBPA (P = 0.0067). The current statistical design specified precisely the most responsive group to phages with chemical adjuvant, namely MRSA FnBPA. Since 6/9 (66.6 %) of MRSA samples studied in the current study produced FnBPA type biofilms, so using phage-based reduction of biofilms with chemical adjuvant seems interesting. Accordingly, the use of anti-MRSA and anti-MSSA phages in the reduction of MRSA and MSSA biofilms, respectively, was shown to be successful especially when coupled with chemical adjuvant helping denature and disintegrate the treated biofilm matrix.

The treated wells in which biofilms were not eliminated completely in the first treatment via phage application with chemical adjuvant were subjected to a second round of treatment. In this instance, the anti-MSSA and anti-MRSA phages, coupled with chemical adjuvant, were applied on the corresponding bacterial biofilms. It was found that all the SA biofilms which were remained after the first round, 3 weak MSSA PIA and 1 weak MSSA FnBPA, were eliminated completely via the second round of phages application with chemical adjuvant. Nevertheless, for MRSA biofilms which were remained after the first round, only 1 weak MRSA PIA and 1 weak MRSA FnBPA biofilm were completely eliminated by the second round of phages application with chemical adjuvant. This indicated the development of resistance by 2/9 (22.2 %) of MRSA biofilms and 2/6 (33.3 %) of FnBPA MRSA biofilms in particular to the attacking phages and this resistance impeded the complete eradication of biofilms in the first and second rounds of phages application.

## Discussion

Microbial biofilms are considered one of the major health problem because they cause chronic infections which are difficult to treat, lead to longer hospitalization time, and can result in much higher treatment costs [29, 31]. Hence, a new approach, rather than the counterproductive antibiotics, must be used. In the current study, interestingly, all the 9 samples of MRSA and 12 samples of MSSA developed biofilms after 24 h. This indicated the highly potent ability of both MSSA and MRSA in developing rapidly biofilms. Moreover, weak production of biofilms in MSSA was half of the all biofilms produced while in MRSA were only 22.2 %. This provided evidence that MRSA production of biofilms is of higher efficacy and of faster pace. Ursic et al. stated that MRSA bacteria have an unrivalled potential of biofilm formation when compared with non-MRSA bacteria [30, 32]. Hence, special care for treating MRSA biofilms is highly needed.

In the current study, we analyzed the formed biofilms of MSSA and MRSA bacteria depending on the nature of the biofilm matrix whether it is PIA/PNAG or proteinacious FnBPA type. FnBPA biofilms in MRSA were found as double frequency as PIA biofilms while in MSSA biofilms the reverse was observed, the frequency of PIA biofilms was double that of FnBPA. These findings are congruous with findings of several previous studies which revealed that protein- mediated biofilm formation capacity seems to be particularly frequent among the highly virulent MRSA isolates, emphasizing the importance of this type of biofilm structure and its capacity for survival and antimicrobial resistance [16, 33]. Therefore, FnBPA type biofilm which is found mainly in highly virulent MRSA can be the key factor for the resilient biofilms produced by MRSA which lay heavy burden on the community medicine, hospital environment, and general health issues. From SEM photos, the proteinacious biofilms were shown with more rigid and compact matrix than PIA biofilms. Hence, the proteinacious FnBPA biofilms of MRSA appear to be difficult for anti-MRSA antibiotic because most of these antibiotics can not get through this compact matrix. In a study, it was found that even a highly effective drug like Vancomycin failed to produce a 2 log reduction in the biofilm embedded MRSA bacterial count and most antiseptics were found unable to completely eliminate MRSA biofilms on plastic or metal surfaces found in hospitals [34].

Accordingly, phage-based approach was taken into consideration for the treatment of MSSA and MRSA biofilms. A previous study revealed that a staphylococcal phage, GH15, was isolated and the endogenous lytic enzyme (LysGH15) was expressed and purified. The lysin LysGH15 displayed a broad lytic spectrum; in vitro treatment killed a number of Staphylococcus aureus strains rapidly and completely, including the methicillin-resistant Staphylococcus aureus (MRSA) [35]. In addition, phages can propagate in their bacterial host and phages produce depolymerases that hydrolyze biofilm extracellular polymers [36] therefore, phage mixtures or engineered phages could provide effective strategies to overcome bacterial biofilms. Lytic bacteriophages could become a new class of anti-biofilm agents. In the current study, when anti-MRSA and anti-MSSA phages without chemical adjuvant were used, findings revealed that phage-based reduction was more remarkable in MSSA than in MRSA and in PIA than in FnBPA; moreover, MRSA FnBPA group was the least responsive to phage-based reduction of biofilms. This showed the difficulty in addressing the problem of MRSA biofilms when phages are used alone. This might be attributed to the limited capacity of phages’ depolymerases to hydrolyze extracellular matrix of MRSA biofilms especially the proteinacious FnBPA type.

As a result of the relatively poor eradication of MRSA and MSSA biofilms by applied phages, it was concluded that the main obstacle is the extracellular matrix of biofilms. Hence, a set of chemical reagents were tried to disrupt the PIA/PNAG or FnBPA protein matrices in parallel with attacking phages to pave the way for phages to access the cell wall of bacterial hosts. After a series of experiments, 0.08 % Benzenthonium chloride and 0.06 % ethanol were used along with applied phages against PIA and FnBPA biofilms, respectively. The resultant mixture of lytic phages and chemical adjuvant granted satisfactory outcomes. After 2 rounds of phages application, 100 % of MSSA PIA and MSSA FnBPA biofilms were removed completely and for MRSA, only 22.2 % of biofilms showed incomplete eradication via resistance against attacking phages with chemical adjuvant. The current findings gave clue to the best way for enabling lytic phages to eradicate efficiently bacterial hosts’ biofilms. Unfortunately, no previous report was found conducting the same principle of phage-chemical biocontrol of MRSA and MSSA. The pioneering findings of the current study imply to new, practical, and cheap approaches to contain, reduce, or eliminate biofilms which are the main refractory source of MRSA and MSSA spread in both hospitals and environment. Moreover, the current phage: chemical mixture can be used in gel base for the prevention of biofilm production on inserted devices into human body such as urinary catheters leading to lowering the use of antimicrobial agents.

The successful control and treatment of MRSA and MSSA biofilms using phages: chemical mixtures have important health advantages. Doolittle et al. [37] demonstrated that the radial movement of T4 phage particles through a biofilm is similar to the process of plaque formation on a lawn of host bacteria, indicating that a single dose of phage could treat a biofilm-related infection. On the contrary, biofilm organisms are tolerant to antimicrobial drugs, disinfectants and biocides [38] and high concentrations of antimicrobials for prolonged treatment durations are normally required to achieve biofilm reduction or eradication [39]. Another drawback of antimicrobial agents is that higher antimicrobial concentrations are required to inactivate or eradicate the biofilm cells as the biofilm ages [40] while this is not found in the case of phages. Moreover, the biofilm extracellular matrix could also affect the efficacy of antimicrobial agents because of diffusion limitations [38] while phages diffuse more easily through alginate gels and extracellular matrix of biofilms. Collectively, all the mentioned advantages of phage-based control of bacterial biofilms when coupled with the currently discovered phage-sparing and biofilm matrix- disrupting chemical agent, the resultant weapon would be so deadly for biofilms of most notorious bugs such as MRSA.

## Conclusions

Taken together, it was concluded that the application of highly lytic anti-MSSA and anti-MRSA phages along with biofilm matrix- disrupting chemicals led to highly successful eradication of host biofilms. The used mixtures of phages: chemical adjuvants are recommended to be used in a large scale evaluation study in order to prepare for the introduction of the current approach of MRSA biofilm control into the routine sanitary procedures done in hospitals. Moreover, the current approach can be used in the prevention of devices-related infections for the in vivo medical instruments, such as urinary catheters.
